# Arbuscular Mycorrhizal Fungi Colonization Promotes Changes in the Volatile Compounds and Enzymatic Activity of Lipoxygenase and Phenylalanine Ammonia Lyase in *Piper nigrum* L. ‘Bragantina’

**DOI:** 10.3390/plants8110442

**Published:** 2019-10-23

**Authors:** Rafaela da Trindade, Laís Almeida, Luciana Xavier, Alba Lúcia Lins, Eloisa Helena Andrade, José Guilherme Maia, Andréa Mello, William N. Setzer, Alessandra Ramos, Joyce Kelly da Silva

**Affiliations:** 1 Programa de Pós-Graduação em Biotecnologia, Universidade Federal do Pará, Belém, PA 66075-110, Brazil; rcabral@ufpa.br (R.d.T.); laisalmeida00@hotmail.com (L.A.); 2 Laboratório de Biotecnologia de Enzimas e Biotransformações, Universidade Federal do Pará, Belém, PA 66075-110, Brazil; lpxavier@ufpa.br; 3 Coordenação de Botânica, Museu Paraense Emílio Goeldi, Belém, PA 66077-830, Brazil; lins@museu-goeldi.br (A.L.L.); eloisa@museu-goeldi.br (E.H.A.); 4 Departamento de Química, Universidade Federal do Maranhão, São Luís, MA 65080-805, Brazil; gmaia@ufpa.br; 5 Instituto de Estudos de Desenvolvimento Agrário Regional, Universidade Federal do Sul e Sudeste do Pará, Marabá, PA 68507-590, Brazil; andreahentz@unifesspa.edu.br; 6 Aromatic Plant Research Center, 230 N 1200 E, Suite 100, Lehi, UT 84043, USA; wsetzer@chemistry.uah.edu; 7 Instituto de Estudos em Saúde e Biológicas, Universidade Federal do Sul e Sudeste do Pará, Marabá, PA 68507-590, Brazil; rezende@unifesspa.edu.br

**Keywords:** black pepper, *Claroideoglomus etunicatum*, *Rhizophagus clarus*, sesquiterpenes

## Abstract

Arbuscular mycorrhizal fungi (AMF) have been used to promote numerous benefits to plants. In this study, we evaluated the symbiosis between AMF species (*Rhizophagus clarus*, *Claroideoglomus etunicatum*) and *Piper nigrum* L. ‘Bragantina’. Volatile compounds, lipoxygenase (LOX) and phenylalanine ammonia-lyase (PAL) activities, and total phenolic content were monitored from 1 to 60 days post-inoculation (dpi). Hyphae, arbuscles, and vesicles were observed during the root colonization. In the leaves, AMF induced an increase of sesquiterpene hydrocarbons (54.0%–79.0%) and a decrease of oxygenated sesquiterpenes (41.3%–14.5%) at 7 dpi and 60 dpi (41.8%–21.5%), respectively. Cubenol, the main volatile compound of leaves, showed a significant decrease at 7 dpi (21.5%–0.28%) and 45 dpi (20.4%–18.42%). β-caryophyllene, the major volatile compound of the roots, displayed a significant reduction at 45 dpi (30.0%–20.0%). LOX increased in the roots at 21, 30, and 60 dpi. PAL was higher in leaves during all periods, except at 60 dpi, and increased at 21 and 45 dpi in the roots. The total phenolic content showed a significant increase only in the roots at 30 dpi. The results suggested that AMF provided changes in the secondary metabolism of *P. nigrum*, inducing its resistance.

## 1. Introduction

Black pepper (*Piper nigrum* L.) is a basal angiosperm, which integrates the magnoliid clade and the family Piperaceae Giseke; it is a perennial, semi-woody, and climbing plant [1]. The plant occurs naturally in the forests of Malabar (India); its center of origin, however, it has been introduced in tropical regions of south and southeast Asia and South America [2]. Owing to its popularity in the culinary industry and broad commercialization in the international market, black pepper is considered the “king of the spices” [3]. Its fruits, resin oil, and essential oil are used as flavouring agents in food preservation, cosmetics, and the perfume industry [4].

Brazil is among the countries with the highest production and export of black pepper, contributing about 17% to international production [5]. In recent years, this index has been displaying oscillations, mainly owing to occurrence of fungal diseases such as fusariosis or root rot (*Fusarium solani* f. sp. *piperis*), yellow wilt (*F. oxysporum*), anthracnose (*Colletotrichum gloeosporioides*), and phythopthora roots (*Phythopthora capsici*) [6,7,8].

Prophylactic measures such as phytosanitation, cultural practices, and chemical treatments are commonly recommended for the management of the diseases of *P. nigrum* [9]. The induced resistance process by growth promoters and antagonist microorganisms has been mentioned in the literature as a viable alternative for the control of plant illness [10], although it is still barely explored for the black pepper crop. In this sense, it becomes relevant to investigate alternative measures of control of diseases that affect black pepper, which are less expensive and accessible to the productive chain of the crop.

About 80% of land plants live in symbiosis with arbuscular mycorrhizal fungi (AMF) [11]. These fungi present an obligate biotrophic lifestyle, and then during the pre-symbiotic phase, the penetration apparatus (PPA) triggers microbe-associated molecular patterns (MAMPs) that mediate suppression of defense-related genes and enable the establishment of colonization [12]. In addition, AMFs contribute to plant development, and activate a mechanism known as mycorrhizal-induced resistance (MIR) [13], which promotes biochemical and anatomical changes in the host tissue and expression of defense genes against pathogens and environmental stresses. 

Mycorrhizal colonization may lead to an increase in the phenolic compounds content and activity of key defense-related enzymes such as lipoxygenases (LOXs) and phenylalanine ammonia-lyase. LOXs are ubiquitous enzymes that play important roles in plants, such as mobilization of stock lipids to initiate seed germination, plant growth, and development, as well as responses to pest and pathogen injury through the production of hydroperoxides from plants, fatty acids, jasmonic acid, volatile aldehydes, and oxyacids [14,15]. PAL acts on the primary and secondary metabolism of higher plants. It is highly regulated during development and lignification. Its activity can be intensified by environmental factors such as low nutrient levels, excess light, and fungal infection [16]. These enzymes have been investigated during the symbiosis with AMFs, owing to their influence on the colonization process and because they constitute biological markers of the plant physiological state [17,18,19].

Root diseases caused by soil-borne pathogens can be reduced by AMF; therefore, they can be used as biocontrol agents [20]. Previous studies reported the AMF effects on *P. nigrum* cultivars: the ‘Cingapura’ cultivar showed better vegetative development after inoculation by *Acaulospora* sp., *Gigaspora heterogama*, *G. margarita,* and *Glomus macrocarpum* [21]. The ‘Guajarina’ cultivar had a lower incidence of fusariosis after inoculation by *Scutellospora gilmorei* [22], and the ‘Bragantina’ cultivar exhibited variations in the chemical compositions of leaf essential oils, with the increase of α-bisabolol (32.3%–48.5%) and elemol (11.4%–23.2%) after inoculation by *Glomus clarum* and *G. etunicatum* [23].

In this work, the ‘Bragantina’ cultivar was selected because of its wide cultivation in northern Brazil and to present high productivity, resistance to yellow wilt, and its susceptibility to fusariosis [24]. Thus, the aim of this study was the evaluation of the pattern of root colonization and changes of volatile profile, phenolic content, and enzymatic activities during the association with AMF.

## 2. Results

### 2.1. Monitoring of Colonization of P. nigrum Roots by AMF

**Figure 1:**
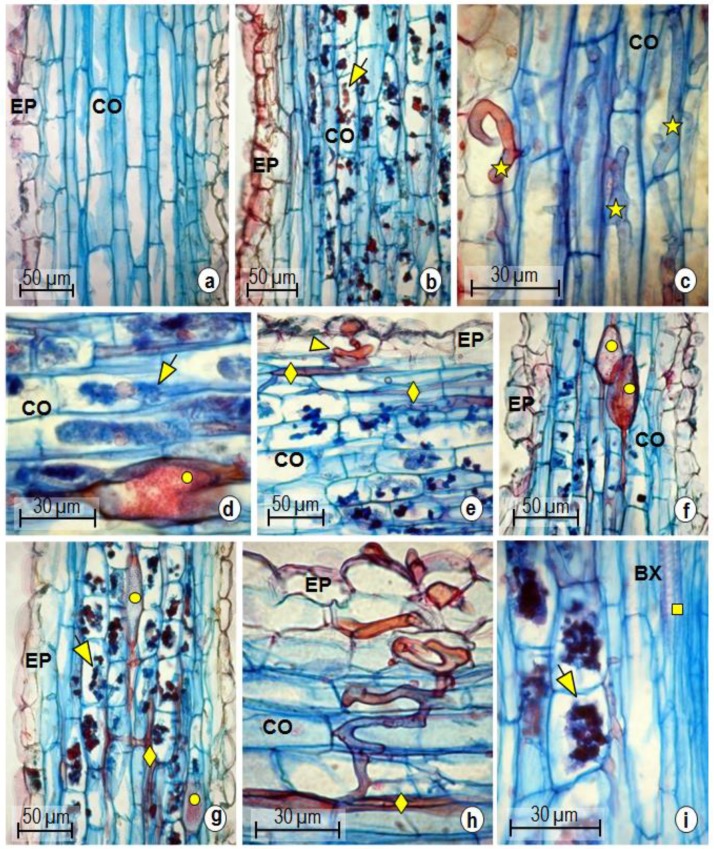
Longitudinal section of *Piper nigrum* roots colonized by arbuscular mycorrhizal fungi. (**a**) Control sample; (**b**) 7 day post inoculation (dpi); (**c,d**) 15 dpi; (**e,f**) 30 dpi; (**g**) 60 dpi; (**h,i**) 90 dpi. EP: epidermis; CO: cortex; BX: bundle of xylem. Symbols: (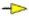): arbuscules; (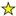): intracellular hyphae; (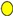): vesicle; (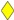): intercellular hyphae; (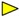): hyphopodium; (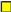): xylem.

The colonization of *P. nigrum* roots was established with the concentration of inoculum used, and mycorrhizal structures were absent in the control group (Figure 1a). At 7 dpi, arbuscles in the cortex of the roots were observed (Figure 1b). At 15 dpi, numerous intracellular hyphae (Figure 1c), terminal arbuscles, and vesicles (Figure 1d) were noted. Arbuscles were visualized near or overlying the plant cell nucleus (Figure 1d). The penetration apparatus (PPA) consisted of hyphopodia, and intracellular hyphae (Figure 1e). Vesicles, and intercellular hyphae (Figure 1f) were noted at 30 dpi. Intense colonization of the radicular cortex with the presence of vesicles, arbuscles, and intracellular hyphae was indicated at 60 dpi (Figure 1g). On the final day of evaluation (90 dpi), fungal entry was still observed in the plant cell (Figure 1h). Intercellular hyphae were predominant in *P. nigrum* roots, characteristic of the Arum type of colonization, comprising extensive intercellular hyphae in the cortical cells, which form terminal arbuscles, connected by hyphal trunks (Figure 1i). Meristematic and vascular tissues were not colonized (Figure 1i).

### 2.2. Variation of Volatile Compounds

**Figure 2:**
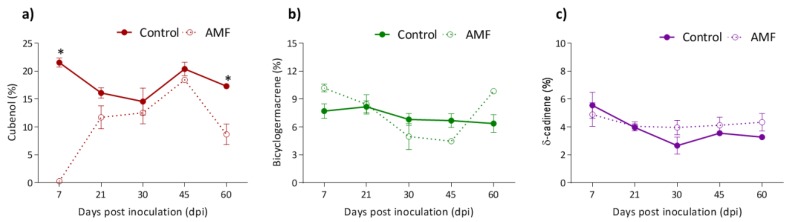
Variation of the main volatile compounds of leaves of *Piper nigrum* in association with arbuscular mychorrhizal fungi. (**a**) cubenol; (**b**) bicyclogermacrene; (**c**) δ-cadinene. Asterisks on the bars represent statistically significant differences between the treatment and control groups, at the 5% probability level, by Bonferroni. AMF, arbuscular mycorrhizal fungi.

The total chemical composition of volatile compounds identified in the leaves was around 98.0%, with sesquiterpene hydrocarbons and oxygenated sesquiterpenoids predominating. AMF association promoted an increase of sesquiterpene hydrocarbons at 7 dpi (54.03%–79.0%). However, the oxygenated sesquiterpenoids contents were reduced in plants inoculated at 7 dpi (41.34%–14.48%) and 60 dpi (41.78%–21.45%) (Table 1). The main volatiles from leaves were cubenol, bicyclogermacrene, and δ-cadinene. There was a significant decrease (*p* < 0.05) in the content of cubenol of inoculated groups at 7 and 60 dpi (21.52%–0.27% and 17.31%–8.67%, respectively) (Figure 2). At 30 dpi, there was an increase in the production of 1-hexanol (0.47%–5.20%), (2*E*)-hexenal (0.46%–1.35%), and hexanal (0.16%–0.53%) in the leaves (Table 1).

**Figure 3:**
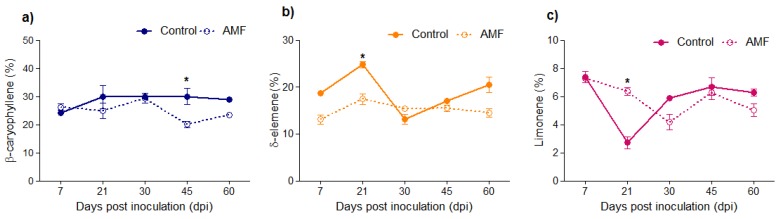
Variation of the main volatile compounds of roots of *Piper nigrum* in association with arbuscular mycorrhizal fungi. (**a**) β-caryophyllene; (**b**) δ-elemene; (**c**) limonene. Asterisks on the bars represent statistically significant differences between the treatment and control groups, at the 5% probability level, by Bonferroni.

In the roots, the main compounds identified were monoterpenes and sesquiterpene hydrocarbons, corresponding to 96% of the total chemical composition (Table 2). The main compound classes did not display essential changes. β-caryophyllene, δ-elemene, and limonene were the major constituents of the roots. β-caryophyllene decreased (*p* < 0.05) at 45 dpi in the inoculated group (30.0%–20.0%) and the content of both δ-elemene and limonene changed (*p* < 0.05) at 21 dpi with AMF. δ-elemene decreased (24.78%–17.45%) and limonene increased (2.71%–6.36%) (Figure 3, Table 2).

### 2.3. Lipoxygenase (LOX) Activity

**Figure 4:**
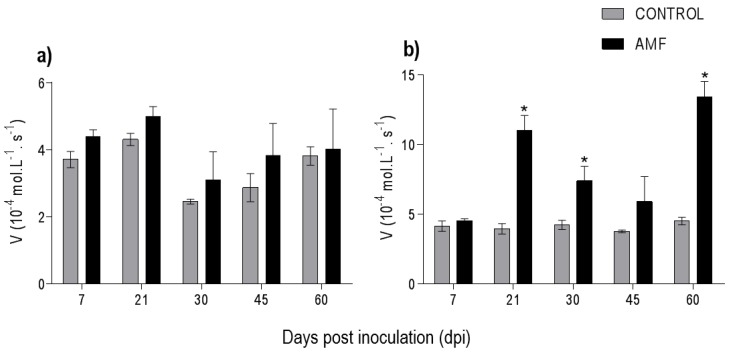
Activity of lipoxygenase in *Piper nigrum* L. (**a**) leaves and (**b**) roots. Asterisks on the bars represent statistically significant differences between the treatment and control groups, at the 5% probability level, by Bonferroni.

During the mycorrhizal colonization of *P. nigrum*, there were no variations in the reaction rate of LOX in the leaves (Figure 4a). However, there was increase of LOX (*p* < 0.05) in the colonized roots at 21, 30, and 60 dpi of 64.2%, 42.7%, and 66.4%, respectively (Figure 4b).

### 2.4. Phenylalanine Ammonia Lyase (PAL) Activity

**Figure 5:**
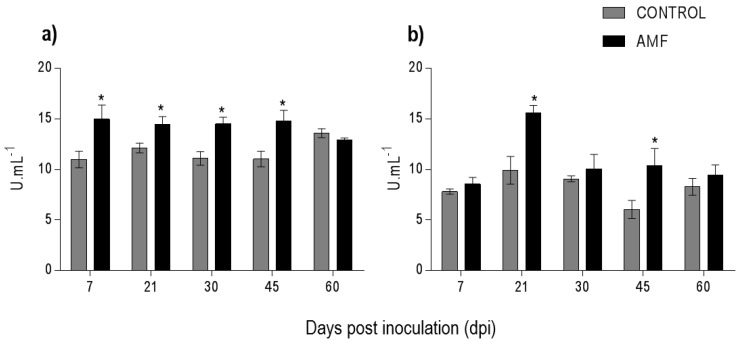
Activity of phenylalanine ammonia-lyase in *Piper nigrum* L. (**a**) leaves and (**b**) roots. Asterisks on the bars represent statistically significant differences between the treatment and control groups, at the 5% probability level, by Bonferroni.

Mycorrhizal colonization induced the highest production of PAL in *P. nigrum* in the leaves and roots. In the leaves, there was a significant difference (*p* < 0.05) in the unit of enzyme/mL of extract at 7 (11.0–15.0); 21 (12.12–14.46); 30 (11.09–14.53), and 45 (11.04–14.78) dpi (Figure 5a). In the roots, there was a greater production of the enzyme at 21 (9.92–15.6) and 45 (6.06–10.4) dpi (Figure 5b).

### 2.5. Total Phenolics Determination

**Figure 6:**
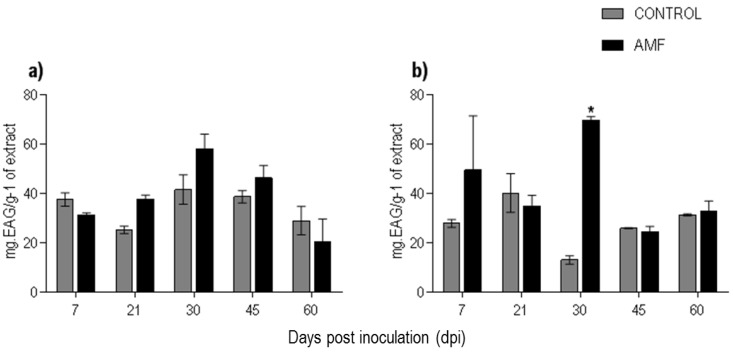
Total phenolic compounds in *Piper nigrum* L. (**a**) leaves and (**b**) roots. Asterisks on the bars represent statistically significant differences between the treatment and control groups, at the 5% probability level, by Bonferroni.

The quantification of the phenolic compounds in the extracts showed variation only in the roots. There was an increase from 13.05 ± 1.66 to 69.59 ± 1.59 mg EAG/g−1 in the colonized roots at 30 dpi (Figure 6a). The amounts of phenolic compounds from the plants inoculated with AMFs did not correlate with the enzymatic activity of PAL in leaves (r^2^ = 0.349) and roots (r^2^ = 0.071).

## 3. Discussion

Among the methods used for the visualization of AMF in roots, clearing and staining with KOH 10% and trypan blue in lactophenol 0.1% is commonly cited in the literature [25]. Our results showed that plant anatomy techniques are effective for characterization of AMF colonization. The root colonization of *P. nigrum* was established at 7 dpi. In most plants, the first stage of establishment of mycorrhizae occurs with the release of exudates by the roots, which attract and induce molecular mechanisms of fungal penetration. Before colonization, a perifungal membrane develops and forms an apoplastic interface between the plant and fungal cell membrane. Then, an invagination of the fungus membrane occurs in the plant cell, forming a thin compartment without changing the integrity of the plant cell. In the apoplastic interface, metabolites are exchanged between symbionts [11]. 

The hyphopod observed (Figure 1e) constitutes part of the PPA, which is fundamental to the pre-symbiotic stage of colonization [26]. Arbuscles (Figure 1d) are temporary structures, undergo turnover, and are essentials in the mycorrhizal interaction [11]. During the formation of the arbuscles, the fungal cell wall becomes less thick, a fragmentation of the vacuole occurs in the plant cell, and the disappearance of amyloplasts and the nuclei from arbuscles overlap with those of the vegetal cell [27]. The PPA (Figure 1e) and vesicles (Figure 1f) that were observed at 30 dpi suggested the continuous entry of AMF into the plant cell, which is probably stimulated by the degeneration of the arbuscles [27]. Vesicles can be formed in the intra and intercellular spaces, are essential storage organs containing lipids with numerous nuclei, and are considered propagation units of the AMF [28]. The analysis of meristematic tissues (Figure 1i) did not display colonization; this occurs as a result of their resistance and constant development [29].

In cultivated species, the predominance of only one colonization type is typical [29]. In *P. nigrum* ‘Bragantina’, however, two types were noted, characterized as an intermediate type, which was previously reported [30]. Experiments have shown that the two types of mycorrhization may occur depending on the host species [31]. Both Arum and Paris types were found in the root systems of *Cucumis sativus* (cucumber) and *Solanum lycopersicum* (tomato) [32]. The occurrence of two colonization patterns in *P. nigrum* may be related to its dependence on mycorrhization [21], which may help in obtaining minerals because the Amazon soils are considered deficient in nutrients [33]. 

Arbuscular mycorrhiza association promoted changes in essential oil composition of the leaves and the roots of *P. nigrum*. The amounts of terpenoids identified in the leaves of *P. nigrum* (Table 1) were similar to a specimen collected in Trivandrum (India), which displayed 63.3% of sesquiterpene hydrocarbons and 32.4% of oxygenated sesquiterpenoids [34]. AMF association increased the sesquiterpene hydrocarbons (54.03%–78.9%) at 7 dpi and reduced oxygenated sesquiterpenoids at 7 dpi (41.34%–14.48%) and at 60 dpi (41.78%–21.45%). However, in a similar study, a decrease of sesquiterpene hydrocarbons (25.4%–10.6%) and an increase of oxygenated sesquiterpenes (67.0%–82.7%) were reported [23]. The association between plants and AMFs can induce numerous variations in terpenoid contents. These molecules have a diverse set of structures and play many roles in plant metabolism, such as hormonal regulation of plant growth and protection against herbivore and pathogen attacks, which should be considered to improve the yield and quality of terpenoids in agricultural crops [35].

Composition, concentration, and mixture of volatile terpenoids promote an indirect effect in the defense against herbivory through changes in the plant’s attractiveness and/or the herbivore’s behavior. *Phaseolus vulgaris* seedlings infested with spider mites and inoculated with *Funneliformis mosseae* emitted β-ocimene and β-caryophyllene in greater quantity, culminating in the attraction of mite predators [36]. 

The occurrence of β-caryophyllene as a major volatile compound in the *P. nigrum* roots has been reported in other studies. The essential oil of *P. nigrum* collected in Haikou (China) was rich in β-caryophyllene, α-humulene (51.2% and 6.76%), and δ-3-carene (6.0%) [37]. Previous studies reported that AMF association had not influenced the β-caryophyllene concentration in *P. nigrum* roots [23]. 

Volatile organic compounds (VOCs) such as 1-hexanol, (2*E*)-hexenal, and hexanal increased in the leaves of inoculated plants (Table 1). This behavior can be a response to biotic stress during the linoleic acid oxidation by LOX action; the aldehyde (3*Z*)-hexenal is the first product formed [38]. AMF colonization also induced an increase in LOX activity (Figure 4). VOCs such as (3*Z*)-hexenol produced from injured plants can promote plant–plant communication responses for the pre-defense of nearby non-attacked plants and may confer protection against insect attack and inhibit colonization by pathogens [39]. 

LOX activity in the roots was higher than in the leaves, probably owing to the inoculation of AMF directly on the soil on the roots. The LOX pathway results in different classes of oxylipins and jasmonic acid (JA). JA plays a vital role in the growth–defense balance, and in the establishment and mutualistic interaction of the host plant and AMF [40,41]. The joint action of LOX and AMF have critical roles in plant development [42]. Colonization by *F. mosseae* and *Rhizophagus irregularis* induced the LOX-9 pathway in roots of *S. lycopersicum* and showed the expression of a gene coding for LOX in leaves, respectively [43,44]. The higher activity of LOX in the roots, observed in the present study, corroborates the hypothesis of Morcillo et al. (2012) [17], which suggests that although AMF performs beneficial actions to the plants, they initially penetrate plant cells, inducing and activating defense and signal transduction pathways such as LOX. During this mechanism, the plant controls the level of colonization of these fungi, aiming to maintain a symbiotically stable relationship. LOXs may also have increased activity under biotic and/or abiotic stimuli, such as by microorganisms, drought, or high-salinity stress [24,44,45,46]. 

Phenylalanine ammonia-lyase is an enzyme widely found in higher plants, and plays a key role in the primary metabolism, acting in the development and lignification in the plants body. Furthermore, in the secondary metabolism, it acts at the beginning of the shikimic acid pathway, and thus is also involved in the protection against biotic and abiotic stresses [47,48]. PAL is the first enzyme and chalcone isomerase the second enzyme that catalyzes the biosynthesis of flavonoids and isoflavonoids, compounds considered chemoattractants for AMF colonization [48]. These enzymes had an increase in their activity during the first stages of colonization of alfalfa roots (*Medicago sativa* L. ‘Cilboa’) by *Glomus intraradix*, and subsequently reduced to levels below the control group [49], suggesting that AMF initiate a defense response in the host, but then suppress this response, with the aim of establishing themselves in the plant’s root tissues [13].

The association of *Lycopersicon esculentum* with *Glomus macrocarpum* and *G. fasciculatum* promoted at least a five-fold increase in PAL activity [50]. In contrast to our results, the inoculation of *Valeriana jatamansi* with *Glomus intradices* induced greater PAL activity in roots than in leaves. Furthermore, there was an increase in the contents of phenolic compounds and tannins [51]. Another study demonstrated an increase of PAL production was efficient in protecting of *P. nigrum* ‘Reyin-1′ (susceptible) and *Piper flaviflorum* (resistant) against the phytopathogenic fungus *Phytophthora capsica*. In addition, histochemical analyses of the stem of these two species showed a greater deposition of lignin in the vascular bundles of resistant plants [52].

Phenolic compounds are essential in the symbiotic interaction between plants and microorganisms because they act as signaling molecules at the beginning of the association and establishment of mycorrhizal colonization and as defense compounds in the plant [53]. Flavonoids present in root exudates play an essential role for the beginning and establishment of symbiosis [54]. Soybean seeds (*Glycine max*) germinated in association with *Glomus fasciculatum* and *G. mosseae* (1:1) presented a greater quantity of phenolic compounds compared with control plants (1.81–2.712 mg gallic acid equivalent (GAE)/g). In addition, the inoculated plants exhibited maximum free radical-scavenging ability compared with the control group, enhancing its nutraceutical potential [55].

The highest production of phenolic compounds in the roots of *P. nigrum* possibly occurred because this organ maintains intrinsic contact with the AMFs. In contrast to the results of this present study, a previous study showed that the inoculation of AMFs in seedlings of *P. nigrum* ‘Bragantina’ did not induce changes in the phenolic contents of roots [23].

## 4. Materials and Methods 

### 4.1. Plant Material

*Piper nigrum* seedlings of the ‘Bragantina’ cultivar were purchased from a local producer from Castanhal (Pará state, Brazil) in February 2016. The plants were acclimatized and maintained in a greenhouse with the daily watering regime. After thirty days, the plants were transplanted to propylene bags, one per pot, with commercial substrate containing a mixture of limestone, castor bean (*Ricinus communis*), bone meal, and expanded vermiculite type B. A voucher of the *P. nigrum* was deposited in the Herbarium of Museu Paraense Emílio Goeldi (MPEG) and was registered as MG224384.

### 4.2. Multiplication of AMF Spores, Production of Inoculum and Inoculation

Arbuscular mycorrhiza spore was obtained from a 50 g sample of soil rhizosphere harvested from the southeast of Pará state (Brazil). The spore’s extraction was performed by wet sieving and decanting method [56] and 40% sucrose centrifugation [57]. AMF identification was done according to its morphological characteristics [58,59] and was propagated in sterile sand, using *Brachiaria brizantha* as trap culture [23]. Inoculum with the proportion of 50% of each fungal species (*Rhizophagus clarus* and *Claroideoglomus etunicatum*), composed of a mixture of spores (density of 90 spores/g soil), hyphae, root fragments, and sterile sand, was used during the inoculation. 

*Piper nigrum* seedlings were removed from bags, pits with approximately 2 cm thick were opened, and 6 g of inoculum was spread superficially on the roots. Finally, the seedlings were replanted. The control group was composed of non-inoculated seedlings.

### 4.3. Experimental Design

Two independent experiments were designed, both arranged in completely randomized blocks. For the chemical profile and enzymatic analyses, thirty plants were separated into two groups: inoculated with AMFs (treatment, 15 plants) and uninoculated plants (control group, 15 plants). The collection of leaves and roots occurred at 7, 15, 21, 30, and 60 days post inoculation (dpi), after which the plants were sacrificed. For anatomical analyses, a total of eighteen plants were employed of these three and fifteen comprised the control and inoculated groups, respectively. The collections of root fragments were performed at 1, 3, 7, 15, 30, 60, and 90 dpi. After 60 dpi, the first plants were reused for analysis. All collections were performed in triplicate.

### 4.4. Mycorrhizal Colonization in *P. nigrum* Roots

For the visualization of mycorrhizal colonization, a qualitative analysis was done based on the presence and absence of fungal structures and the development of colonization. Usual techniques in plant anatomy were employed [60]. Root fragments of approximately 1 cm were fixed during 24 h in FAA70 (formaldehyde, acetic acid, and ethanol 70%, 1:1:1, v/v). Afterward, the samples were dehydrated with a series of butyl alcohol treatments and embedded in histological paraffin [61]. Longitudinal sections (12–18 μm) were obtained using an automatic microtome (Leica^®^ RM 2245, Nussloch, Germany), stained with Astra blue and Safranine [62] and mounted in Entellan^®^ resin. Photomicrographs were obtained using a Canon digital camera model A65015 coupled to a Zeiss microscope model 426126.

### 4.5. Extraction and Analysis of the Essential Oils

The essential oils fractions from fresh leaves and roots (2.0 g) of *P. nigrum* were obtained by simultaneous distillation–extraction process using a Likens–Nickerson apparatus for 2 h and *n*-pentane (3 mL) as solvent. After extraction, an aliquot (1.0 μL) of the organic phase was analyzed by gas chromatography. Qualitative analysis was carried out on a Gas Chromatography-Mass Spectrometry (GC-MS) (Shimadzu QP2010 plus instrument) under the following conditions: Rtx-5MS silica capillary column (30 m × 0.25 mm film thickness); programmed temperature, 60–240 °C (3 °C/min); injector temperature, 200 °C; carrier gas, helium, adjusted to a linear velocity of 1.2 mL/min; injection type, splitless; split flow was adjusted to yield a 20:1 ratio; septum sweep was a constant 10 mL/min; EIMS, electron energy, 70 eV; and temperature of the ion source and connection parts, 200 °C. The retention indices were calculated for all the volatile constituents using a homologous series of *n*-alkanes (C8–C32, Sigma-Aldrich) [63]. The identification of compounds was performed by comparison of mass spectra and retention indices with data present in the libraries of Adams, National Institute of Standards and Technology (NIST), and Flavour and Fragrance Natural and Synthetic Compounds (FFNSC2) [63,64,65]. The component percentages are based on peak integrations without standardization.

### 4.6. Lipoxygenase (LOX) Activity

Leaves and roots were collected and macerated in liquid nitrogen, then the mixture of 1 g of powder plus 3 mL of sodium phosphate buffer (50 mM, pH 6.5) was centrifuged, and the supernatant was used as a source of enzymes. Linoleic acid (78 μL, Sigma-Aldrich, USA) and Tween 20 (90 μL, Sigma-Aldrich) were mixed with boiling water (10 mL) and a few drops of sodium hydroxide (0.5 N), in order to prepare the substrate. The final volume was adjusted to 25 mL, resulting in a sodium linoleate solution (10 mM), which was stored at −20 °C. The test was performed with 5 μL of source of enzymes, 50 μL of sodium linoleate (10 mM), and 1945 μL of sodium phosphate buffer. The mixture was read in a UV/visible spectrophotometer at 234 nm, the increase in absorbance indicated the presence of a conjugated double-bond system in the formed hydroperoxide. LOX activity was determined by monitoring the change in absorbance for 120 seconds, using the specific molar extinction coefficient of 25,000 L^−1^.cm^−1^ moles for calculations [66,67].

### 4.7. Phenylalanine Ammonia Lyase (PAL) Activity

Leaves and roots were submitted to maceration in liquid nitrogen to obtain a powder. Each sample (1.0 g) was homogenized in 2 mL of sodium borate buffer (0.3 mM, pH 8.8), 1 mM ethylenediaminetetraacetic acid (EDTA), 1 mM dithiothreitol (DTT), and 5% polyvinylpolypyrrolidone (PVP). After centrifugation, an aliquot of the supernatant (0.5 mL) was mixed with 1.0 mL of reaction buffer containing sodium borate 0.3 mM, pH 8.8, and 0.03 mM L-phenylalanine. The reaction was incubated for 15 min at room temperature, and then the absorbance was read at 290 nm in the UV/visible spectrophotometer. Enzyme units (U) were calculated using a specific molar extinction coefficient of 9630 mol.L^−1^.cm^−1^. One U was defined as the amount of enzyme that catalyzes the conversion of 1 μmol.min^−1^ of L-phenylalanine substrate to *trans*-cinnamic acid per minute, under the specific assay conditions, and volumetric activity was obtained by U.mL^−1^. The experiments were performed in a series of three repetitions each [68].

### 4.8. Folin–Ciocalteu Total Phenolics Determination

Fresh leaves and roots (2 g) were extracted by percolation (96 h) with 50 mL of ethyl acetate. After solvent evaporation, the total phenolics concentration was determined using the Folin–Ciocalteu method [69]. An aliquot of 500 μL of extract in methanol (20 mg.mL^−1^) was used to react with 250 μL of reagent (1 N) and 1250 μL of sodium carbonate (75 g L^−1^). After 30 min incubation in the dark, the absorbance of the mixture was read at 760 nm using a UV/visible spectrophotometer. The experimental calibration curve was prepared using gallic acid at concentrations of 0.5 to 10.0 mg L^−1^ and the content of total phenolics was expressed as gallic acid equivalents (GAE) in milligrams per gram of extract (mg/GAE g^−1^).

### 4.9. Statistical Analysis

All analyses were performed in triplicate, compared with the control group, and the data were expressed as means ± standard deviation. GraphPad 6.0 software was used. Analysis of variance was conducted by Bonferroni test following two-way analysis of variance (ANOVA), differences at *p* < 0.05 were considered statistically significant. Pearson’s correlation coefficient (*p* < 0.05) was applied to verify the relationship between PAL activity and total phenolic content.

## 5. Conclusions

The symbiotic association of AMF established in *P. nigrum* is manifested in the production of volatile compounds related to plant defense, among them, the constituents of the enzyme lipoxygenase pathway, as well as limonene, which was produced in more significant amounts in the roots. These results suggest that the association of *P. nigrum* with AMFs may serve to induce the production of defense compounds, which can help it alleviate biotic and abiotic stresses and may serve as a biological control of black pepper disease.

## Figures and Tables

**Table 1:** Comparison of volatile compounds produced in *Piper nigrum* leaves non-inoculated and inoculated with arbuscular mycorrhizal fungi (AMF) (mean ± standard deviation).

Compounds	RIL	RIC	7 dpi		21 dpi		30 dpi		45 dpi		60 dpi	
			CONTROL	AMF	CONTROL	AMF	CONTROL	AMF	CONTROL	AMF	CONTROL	AMF
*n*-hexanol	863	857	0.09 ± 0.16		0.35 ± 0.60		0.47 ± 0.66	5.20 ± 4.51	0.78 ± 0.50	1.00 ± 1.41	1.71 ± 1.42	1.73 ± 1.02
δ-elemene	1335	1331	4.08 ± 0.41	4.42 ± 1.65	4.59 ± 0.56	4.81 ± 0.83	3.81 ± 0.60	2.54 ± 0.77	3.58 ± 0.80	2.55 ± 0.06	4.08 ± 0.94	4.84 ± 0.36
α-cubebene	1345	1344	3.41 ± 0.9	3.54 ± 1.65	4.02 ± 0.49	3.82 ± 0.54	2.91 ± 0.56	2.60 ± 1.02	3.03 ± 0.33	2.41 ± 0.10	3.00 ± 0.58	5.24 ± 0.69
α-copaene	1374	1373	4.32 ± 2.25	3.85 ± 1.8	5.19 ± 0.80	4.84 ± 0.96	3.91 ± 1.15	2.74 ± 1.17	3.81 ± 0.65	2.49 ± 0.42	3.50 ± 0.90	6.42 ± 1.45
β-cubebene	1387	1385	2.53 ± 0.4	4.62 ± 2.75	3.04 ± 0.91	3.84 ± 2.02	2.18 ± 0.30	2.53 ± 2.34	2.57 ± 1.03	3.29 ± 0.28	3.50 ± 0.31	4.55 ± 0.71
α-gurjunene	1409	1410	3.82 ± 1.39	2.98 ± 1.3	5.13 ± 0.89	4.58 ± 0.65	3.59 ± 0.45	2.28 ± 0.77	3.53 ± 0.95	2.16 ± 0.20	3.61 ± 0.85	5.82 ± 1.54
β-caryophyllene	1417	1417	4.63 ± 1.72	5.74 ± 2.73	4.81 ± 0.83	4.97 ± 1.73	3.86 ± 0.90	2.82 ± 1.30	3.33 ± 0.41	3.07 ± 1.53	3.46 ± 1.02	6.28 ± 1.12
β-selinene	1489	1486	5.36 ± 2.29	5.19 ± 2.01	5.91 ± 1.02	6.85 ± 2.79	6.21 ± 2.52	3.48 ± 1.44	4.46 ± 1.03	2.88 ± 0.32	4.10 ± 1.07	5.69 ± 0.64
*trans*-muurola-4(14),5-diene	1493	1488	3.13 ± 0.37	3.46 ± 1.16	3.06 ± 0.41	1.99 ± 1.73	1.00 ± 1.41	1.39 ± 1.26	2.46 ± 0.24	2.17 ± 0.06	2.45 ± 0.52	3.08 ± 0.06
bicyclogermacrene	1500	1493	7.70 ± 1.34	10.1 ± 0.59	8.16 ± 1.11	8.43 ± 1.81	6.80 ± 0.91	4.96 ± 1.99	6.68 ± 1.28	4.46 ± 0.30	6.36 ± 1.64	9.83 ± 0.35
α-muurolene	1500	1496	3.27 ± 1.33	2.93 ± 0.94	4.10 ± 0.48	3.94 ± 0.81	2.80 ± 0.93	2.65 ± 1.56	2.72 ± 0.39	1.93 ± 0.45	2.66 ± 0.80	3.31 ± 0.13
cubebol	1514	1513	0.33 ± 0.57	0.99 ± 1.71	2.00 ± 2.35	0.20 ± 0.35	2.62 ± 3.70	2.48 ± 2.26	3.29 ± 1.02	0.93 ± 1.31	3.92 ± 2.35	2.62 ± 0.17
δ-cadinene	1522	1515	5.54 ± 1.62	4.88 ± 1.19	3.96 ± 0.42	4.04 ± 0.57	2.66 ± 0.86	3.96 ± 0.90	3.55 ± 0.34	4.12 ± 0.83	3.27 ± 0.11	4.33 ± 0.89
caryophyllene oxide	1582	1584	3.73 ± 1.10	4.71 ± 0.86	2.36 ± 0.11	3.12 ± 0.97	4.92 ± 0.23	5.00 ± 1.94	3.43 ± 0.42	4.93 ± 0.85	3.84 ± 0.66	2.07 ± 0.15
*epi*-cubenol	1627	1624	0.86 ± 0.58	0.95 ± 1.18	0.30 ± 0.31	0.54 ± 0.47	1.16 ± 1.03	2.21 ± 2.03	1.31 ± 0.20	1.16 ± 0.83	1.05 ± 0.53	1.01 ± 0.04
α-muurolol	1644	1639	2.72 ± 1.15	2.92 ± 1.76	2.03 ± 0.95	2.01 ± 0.79	1.82 ± 0.62	2.20 ± 1.02	3.19 ± 0.18	3.41 ± 1.15	2.70 ± 0.19	1.56 ± 0.26
cubenol	1645	1643	21.53 ± 9.7 *	0.28 ± 0.23 *	16.09 ± 1.3	11.7 ± 2.91	14.55 ± 3.42	12.53 ± 2.81	20.39 ± 1.71	18.42 ± 0.27	17.31 ± 0.24 *	8.67 ± 2.60 *
(2*E*,6*Z*)-farnesal	1713	1706	2.3 ± 0.8	2.1 ± 0.5	0.63 ± 0.09	1.08 ± 0.33	1.02 ± 0.40	1.46 ± 0.33	1.44 ± 0.61	1.48 ± 0.20	1.31 ± 0.47	0.13 ± 0.11
(2*E*,6*E*)-farnesal	1740	1733	3.0 ± 1.3	2.9 ± 1.3	0.94 ± 0.12	1.46 ± 0.43	1.07 ± 0.35	1.84 ± 0.33	1.52 ± 0.53	1.91 ± 0.15	1.52 ± 0.39	0.21 ± 0.18
Monoterpene hydrocarbons			0.6 ± 0.3	0.7 ± 0.9	0.9 ± 0.2	0.6 ± 0.5	0.7 ± 0.2	2.2 ± 1.6	1.0 ± 0.5	1.2 ± 1.0	0.6 ± 0.6	0.4 ± 0.2
Oxygenated monoterpenes			1.5 ± 1.6	1.2 ± 0.9	0.7 ± 0.4	0.7 ± 0.3	0.9 ± 0.5	3.0 ± 1.9	1.0 ± 0.4	1.3 ± 0.6	0.6 ± 0.7	1.3 ± 0.2
Sesquiterpene hydrocarbons			54.0 ± 1.0 *	78.9 ± 3.2 *	70.1 ± 12.2	68.3 ± 19.0	55.2 ± 12.1	44.2 ± 23.7	53.5 ± 11.0	45.4 ± 9.3	52.2 ± 13.4	73.3 ± 10.1
Oxygenated Sesquiterpenes			41.3 ± 1.8 *	14.4 ± 3.8 *	26.1 ± 7.9	26.0 ± 17.2	40.2 ± 18.2	41.2 ± 23.3	41.3 ± 9.5	46.9 ± 14.8	41.7 ± 9.3 *	21.4 ± 4.8 *
Phenylpropanoids			0.0	0.0	0.0	0.0	0.0	0.0	0.0	0.0	0.0	0.0
Others			0.7 ± 0.9	3.1 ± 2.9	1.7 ± 1.4	2.2 ± 1.4	1.3 ± 1.0	7.9 ± 6.8	1.7 ± 1.0	2.8 ± 1.9	3.4 ± 2.2	3.5 ± 1.3
Total			98.1 ± 5.6	98.3 ± 11.7	99.6 ± 22.1	97.9 ± 38.6	98.5 ± 32.1	98.8 ± 57.5	98.7 ± 22.7	97.7 ± 27.8	98.7 ± 26.4	99.9 ± 16.8

This table contains only volatile compounds above 2% present in at least one of the treatments. **RIL**: retention index of library; **RIC**: retention index calculated; **control**: *Piper nigrum* non-inoculated with AMF; **AMF**: *Piper nigrum* inoculated with AMF; dpi: days post inoculation; * statistical difference according to Bonferroni test (*p* < 0.05).

**Table 2:** Comparison of volatile compounds produced in *Piper nigrum* roots non-inoculated and inoculated with arbuscular mycorrhizal fungi (AMF) (mean ± standard deviation).

Compounds	RIL	RIC	7 dpi		21 dpi		30 dpi		45 dpi		60 dpi	
			CONTROL	AMF	CONTROL	AMF	CONTROL	AMF	CONTROL	AMF	CONTROL	AMF
α-thujene	924	917		1.42 ± 2.47	0.94 ± 1.45	2.8 ± 1.53			2.42 ± 1.06	2.91 ± 1.34	2.9 ± 0.73	2.91 ± 0.50
α-pinene	932	934	3.42 ± 0.96	5.24 ± 3.21	4.08 ± 3.26	8.27 ± 4.66	2.09 ± 1.25	2.34 ± 0.82	6.10 ± 3.98	8.4 ± 2.95	8.88 ± 1.94	8.25 ± 1.24
camphene	946	956	7.82 ± 3.63	5.92 ± 5.28		0.01 ± 0.02	5.27 ± 2.55	6.74 ± 0.32			0.02 ± 0.03	0.01 ± 0.02
sabinene	969	970		1.93 ± 3.29	1.54 ± 1.23	2.47 ± 1.24	0.01 ± 0.02	0.01 ± 0.02	2.84 ± 1.31	2.49 ± 1.17	2.88 ± 0.25	2.49 ± 0.64
β-pinene	974	976	4.17 ± 1.11	3.2 ± 0.82	0.32 ± 0.55	1.08 ± 0.61	2.33 ± 1.15	2.36 ± 1.13	1.01 ± 0.55	1.16 ± 0.6	1.30 ± 0.32	1.19 ± 0.19
limonene	1024	1028	8.73 ± 2.38	8.71 ± 2.44	3.7 ± 1.82 *	5.3 ± 1.87 *	5.02 ± 1.52	4.9 ± 1.49	5.65 ± 2.03	5.38 ± 1.70	6.08 ± 0.52	4.95 ± 0.95
camphor	1141	1146	4.8 ± 2.22	4.49 ± 0.85	3.07 ± 1.09	5.28 ± 1.10	4.86 ± 1.89	5.30 ± 0.27	3.68 ± 1.74	7.00 ± 2.6	3.68 ± 0.85	4.31 ± 1.36
isoborneol	1155	1162	1.98 ± 1.2	2.85 ± 0.86	0.49 ± 0.43	1.88 ± 0.32	2.92 ± 0.89	1.40 ± 1.21	1.58 ± 1.64	2.06 ± 1.26	1.62 ± 2.10	2.20 ± 1.08
3,5-dimethoxytoluene		1264	2.2 ± 0.32	1.44 ± 0.28			2.37 ± 0.85	1.79 ± 0.57				
δ-elemene	1335	1333	18.95 ± 0.55	13.29 ± 2.0	24.78 ± 1.23 *	16.75 ± 2.35 *	13.13 ± 2.06	15.7 ± 0.69	19.18 ± 3.61	15.28 ± 1.56	18.10 ± 4.74	15.85 ± 2.63
β-elemene	1389	1387	2.23 ± 0.42	1.9 ± 0.30	2.05 ± 0.86	2.15 ± 0.13	2.49 ± 0.4	2.1 ± 0.30	2.238 ± 0.08	2.29 ± 0.12	2.21 ± 0.163	2.39 ± 0.22
β-caryophyllene	1417	1417	24.15 ± 1.32	26.27 ± 1.66	29.81 ± 5.97	24.9 ± 3.96	29.82 ± 0.29	29.37 ± 2.51	29.9 ± 4.0 *	20.0 ± 1.64 *	28.97 ± 1.08	23.50 ± 0.63
α-humulene	1452	1453	2.6 ± 0.55	2.93 ± 0.50	2.61 ± 0.48	2.66 ± 0.42	2.88 ± 0.30	2.78 ± 0.15	2.898 ± 0.45	2.59 ± 0.27	2.56 ± 0.020	2.69 ± 0.25
α-muurolene	1500	1496	2.81 ± 0.68	2.21 ± 0.52	3.29 ± 1.06	2.62 ± 0.35	3.17 ± 0.96	2.42 ± 0.12	3.098 ± 0.82	2.43 ± 0.36	2.54 ± 0.12	2.60 ± 0.30
β-bisabolene	1505	1505	2.09 ± 0.4	2.02 ± 0.20	2.40 ± 1.30	2.55 ± 1.65	2.31 ± 1.35	1.41 ± 0.40	2.128 ± 1.13	2.41 ± 0.93	2.54 ± 2.07	1.40 ± 0.21
7-*epi*-α-selinene	1520	1515	1.97 ± 0.58	1.58 ± 0.44	1.98 ± 0.58	1.97 ± 0.12	2.24 ± 0.35	1.87 ± 0.25	2.058 ± 0.29	2.04 ± 0.19	1.78 ± 0.29	2.02 ± 0.28
Monoterpene hydrocarbons			25.89 ± 8.59	27.48 ± 18.40	10.59 ± 8.35	19.71 ± 9.95	15.69 ± 7.05	17.13 ± 4.50	18.04 ± 8.95	20.36 ± 7.86	22.08 ± 3.81	19.81 ± 3.57
Oxygenated monoterpenes			9.89 ± 4.17	9.93 ± 2.27	4.33 ± 1.89	8.41 ± 1.76	8.83 ± 3.21	7.30 ± 1.92	5.98 ± 3.94	10.11 ± 4.67	6.44 ± 3.38	7.67 ± 2.78
Sesquiterpene hydrocarbons			62.95 ± 8.12	58.09 ± 9.71	79.02 ± 28.36	63.12 ± 12.72	63.49 ± 16.65	66.27 ± 14.89	70.34 ± 13.55	58.94 ± 15.01	62.46 ± 12.95	57.79 ± 6.08
Oxygenated Sesquiterpenes			0.97 ± 0.32	2.07 ± 0.37	1.36 ± 1.47	4.26 ± 2.75	6.81 ± 5.63	4.31 ± 2.50	1.87 ± 1.35	4.60 ± 3.45	3.55 ± 2.37	5.48 ± 2.82
Phenylpropanoids					0.08 ± 0.09	0.03 ± 0.05	0.06 ± 0.05	0.03 ± 0.05	0.02 ± 0.03	0.06 ± 0.06	0.07 ± 0.09	0.09 ± 0.05
Others			0.22 ± 0.09	0.31 ± 0.09	1.83 ± 0.73	0.98 ± 0.73	2.85 ± 1.32	3.03 ± 1.91	0.97 ± 1.20	0.84 ± 1.20	1.03 ± 1.28	0.96 ± 0.85
Total			99.92 ± 21.29	97.88 ± 30.83	97.21 ± 41.35	96.51 ± 27.95	97.73 ± 33.90	98.07 ± 25.76	97.22 ± 29.02	94.91 ± 32.26	95.63 ± 23.89	91.8 ± 16.14

This table contains only volatile compounds above 2% present in at least one of the treatments. **RIL**: retention index of library; **RIC**: retention index calculated; **control**: *Piper nigrum* non-inoculated with AMF; **AMF**: *Piper nigrum* inoculated with AMF; dpi: days post inoculation; * statistical difference according to Bonferroni test (*p* < 0.05).

## References

[1] Krishnamurthy K.S., Parthasarathy V.A., Saji K.V., Krishnamoorthy B. (2010). Ideotype concept in black pepper (*Piper nigrum* L.). J. Spices Aromat. Crops.

[2] Hao C., Fan R., Ribeiro M.C., Tan L., Wu H., Yang J., Zheng W., Huan Y. (2012). Modeling the Potential Geographic Distribution of Black Pepper (*Piper nigrum*) in Asia Using GIS Tools. J. Integr. Agric..

[3] Srinivasan K. (2007). Black Pepper and its Pungent Principle-Piperine: A Review of Diverse Physiological Effects. Crit. Rev. Food Sci. Nutr..

[4] Nair K.P.P. (2011). The Agronomy and Economy of Black Pepper (*Piper nigrum* L.)—The “King of Spices”. Agronomy and Economy of Black Pepper and Cardamom the “King” and “Queen” of Spices.

[5] Hena M. (2016). Export marketing of pepper: Opportunities and constraints. Asia Pac. J. Res..

[6] Sarma Y.W., Manohara D., Premkuma T.T., Eapen S.J. (2013). Diseases and Insect Pests of Black Pepper (Piper nigrum L).

[7] LSPA (2017). Levantamento sistemático da produção agrícola.

[8] Tremacoldi C.R. (2010). Principais doenças fúngicas da pimenteira-do-reino no Estado do Pará e recomendações de controle.

[9] ICAR—Indian Institute of Spices Research (2015). Black Pepper.

[10] Pieterse C.M.J., Zamioudis C., Berendsen R.L., Weller D.M., Van Wees S.C.M., Bakker P.A.H.M. (2014). Induced systemic resistance by beneficial microbes. Annu. Rev. Phytopathol..

[11] Bonfante P., Genre A. (2010). Mechanisms underlying beneficial plant-fungus interactions in mycorrhizal symbiosis. Nat. Commun..

[12] Recorbet G., Abdallah C., Renaut J., Wipf D., Dumas-Gaudot E. (2013). Protein actors sustaining arbuscular mycorrhizal symbiosis: Underground artists break the silence. New Phytol..

[13] Pozo M.J., Azcón-Aguilar C. (2007). Unravelling mycorrhiza induced resistance. Curr. Opin. Plant Biol..

[14] Siedow J.N. (1991). Plant lipoxygenase: Structure and function. Annu. Rev. Plant Physiol. Plant Mol. Biol..

[15] Feussner I., Wasternack C. (1998). Lipoxygenase catalyzed oxygenation of lipids. Lipid/Fett.

[16] Taiz L., Zeiger E. (2013). Plant Physiology.

[17] Morcillo R.J.L., Ocampo J.A., Garrido J.M.G. (2012). Plant 9-lox oxylipin metabolism in response to arbuscular mycorrhiza. Plant Signal. Behav..

[18] Babenko L.M., Shcherbatiuk M.M., Skaterna T.D., Kosakivska I.V. (2017). Lipoxygenases and their metabolites in formation of plant stress tolerance. Ukr. Biochem. J..

[19] Feussner I., Wasternack C. (2002). The lipoxygenase pathway. Annu. Rev. Plant Biol..

[20] Tahat M.M., Kamaruzaman S., Othman R. (2010). Mycorrhizal Fungi as a Biocontrol Agent. Plant Pathol. J..

[21] Olivera E., Souza P., Matos A.O. (1984). Endomicorrizo dependência da pimenta-do-reino. Fitopatol. Bras..

[22] Chu E.Y., Endo T., Stein R.L.B., Albuquerque F.C. (1997). Avaliação da inoculação de fungos micorrízicos arbusculares sobre a incidência da fusariose da pimenta-do-reino. Fitopatol. Bras..

[23] Da Luz S.F.M., Reis L.A., Lemos O.F., Maia J.G.S., Mello A.H., Ramos A.R., da Silva J.K.R. (2016). Effect of arbuscular mycorrhizal fungi on the essential oil composition and antioxidant activity of black pepper (*Piper nigrum* L.). Int. J. Appl. Res. Nat. Prod..

[24] Castro G.L.S., Lemos O.F., Tremacoldi C.R., Moraes F.K.C., Santos L.R.R., Pinheiro H.A. (2016). Susceptibility of in vitro black pepper plant to the filtrate from a *Fusarium solani* f. sp. *piperis* culture. Plant Cell Tissue Organ. Cult..

[25] Phillips J.M., Hayman D.S. (1970). Improved procedures for clearing roots and staining parasitic and vesicular-arbuscular mycorrhizal fungi for rapid assessment of infection. Trans. Br. Mycol. Soc..

[26] Smith F.A., Smith S.E. (1997). Structural diversity in (vesicular)-arbuscular mycorrhizal symbioses. New Phytol..

[27] Kiriachek S.G., Azevedo L.C.B., Peres L.E.P., Lambais M.R. (2009). Regulação do desenvolvimento de micorrizas arbusculares. Rev. Bras. Cienc. Solo.

[28] Smith S.E., Read D.J. (2008). Colonization of roots and anatomy of arbuscular. Mycorrhizal Symbiosis.

[29] Genre A., Bonfante P. (2005). Building a mycorrhizal cell: How to reach compatibility between plants and arbuscular mycorrhizal fungi. J. Plant Interact..

[30] Muthukumar T., Tamilselvi V. (2010). Occurrence and morphology of endorhizal fungi in crop species. Trop. Subtrop. Agroecosyst..

[31] Dickson S. (2004). The Arum-Paris continuum of mycorrhizal symbioses. New Phytologist..

[32] Kubota M., McGonigle T.P., Hyakumachi M. (2005). Co-occurrence of Arum- and Paris-type morphologies of arbuscular mycorrhizae in cucumber and tomato. Mycorrhiza.

[33] Moreira A., Fageria N.K., Garcia A., Garcia Y. (2011). Soil Fertility, Mineral Nitrogen, and Microbial Biomass in Upland Soils of the Central Amazon under Different Plant Covers. Commun. Soil Sci. Plant Anal..

[34] Sasidharan I., Menon A.N. (2010). Comparative chemical composition and antimicrobial activity of berry and leaf essential oils of *Piper nigrum* L.. Int. J. Biol. Med. Res..

[35] Welling M.T., Liu L., Rose T.J., Waters D.L.E., Benkendorff K. (2015). Arbuscular mycorrhizal fungi: Effects on plant terpenoid accumulation. Plant Biol..

[36] Sharma E., Anand G., Kapoor R. (2017). Terpenoids in plant and arbuscular mycorrhiza-reinforced defense against herbivorous insects. Ann. Bot..

[37] Ao P., Hu S., Zhao A. (1998). Essential oil analysis and trace element study of the roots of *Piper nigrum* L.. Zhongguo Zhong Yao Za Zhi.

[38] Pinto-Zevallos D.M., Martins C.B.C., Pellegrino A.C., Zarbin P.H.G. (2013). Volatile organic compounds in induced plant defense against herbivorous insects. Quím. Nova.

[39] Wei J., Kang L. (2011). Roles of (Z)-3-hexenol in plant-insect interactions. Plant Signal. Behav..

[40] Ponzio C., Gols R., Pieterse C.M.J., Ponzio M.D. (2013). Ecological and phytohormonal aspects of plant volatile emission in response to single and dual infestations with herbivores and phytopathogens. Funct. Ecol..

[41] Yan C., Xie D. (2015). Jasmonate in plant defense: Sentinel or double agent?. Plant Biotechnol. J..

[42] Azcón-Aguilar C., Barea J.M. (1996). Arbuscular mycorrhizas and biological control of soil-borne plant pathogens. An overview of the mechanisms involved. Mycorrhiza.

[43] Feussner I., Kuhn H., Wasternack C. (2001). Lipoxygenase-dependent degradation of storage lipids. Trends Plant Sci..

[44] Cervantes-Gámez R.G., Bueno-Ibarra M.A., Cruz-Mendívil A., Calderón-Vázquez C.L., Ramírez-Douriet C.M., Maldonado-Mendoza I.E., Villalobos M.A., Valdez A., Lopez-Meyer M. (2015). Arbuscular mycorrhizal symbiosis-induced expression changes in *Solanum lycopersicum* leaves revealed by RNA-seq analysis. Plant Mol. Biol. Rep..

[45] Silva M.D., Oliveira M.G.A., Lanna A.C., Pires C.V., Piovesan N.D., Jose I.C., Batista R.B., De Barros E.G., Moreira M.A. (2001). Caracterização da via das Lipoxigenases em plantas de soja resistente e susceptível a *Diaphorte phaseolorum* f. sp. meridionalis, agente causal do cancro-da-haste. Rev. Bras. Fisiol. Veg..

[46] Lim C.W., Han S., Hwang I.S., Kim D.S., Hwang B.K., Lee S.C. (2015). The Pepper Lipoxygenase CaLOX1 plays a role in osmotic, drought and high salinity stress response. Plant Cell Physiol..

[47] MacDonald M.J., D’Cunha G.B. (2007). A modern view of phenylalanine ammonia lyase. Biochem. Cell Biol..

[48] Osakabe Y., Nishikubo N., Osakabe K. (2007). Phenylalanine ammonia-lyase in woody plants: A key swich of carbon accumulation in biomass. Jpn. J. Plant Sci..

[49] Volpin H., Elkind Y., Okon Y., Kapulnik Y. (1994). A vesicular arbuscular mycorrhizal fungus (*Glomus intraradix*) induces a defense response in alfalfa roots. Plant Physiol..

[50] Kapoor R. (2008). Induced resistance in mycorrhizal tomato is correlated to concentration of jasmonic acid. Online J. Biol. Sci..

[51] Jugran A.K., Bahukhandi A., Dhyani P., Bhatt I.D., Rawal R.S., Nandi S.K., Palni L.M.S. (2015). The effect of inoculation with mycorrhiza: AM on growth phenolics tannins phenolic composition and antioxidant activity in *Valeriana jatamansi* Jones. J. Soil Sci. Plant Nutr..

[52] Hao C., Xia Z., Fan R., Tan L., Hu L., Wu B., Wu H. (2016). De novo transcriptome sequencing of black pepper (*Piper nigrum* L.) and an analysis of genes involved in phenylpropanoid metabolism in response to *Phytophthora capsici*. BMC Genom..

[53] Mandal S.M., Chakraborty D., Dey S. (2010). Phenolic acids act as signaling molecules in plant-microbe symbioses. Plant Signal. Behav..

[54] Steinkellner S., Lendzemo V., Langer I., Schweiger P., Khaosaad T., Toussaint J.P., Vierheilig H. (2007). Flavonoids and strigolactones in root exudates as signals in symbiotic and pathogenic plant-fungus interactions. Molecules.

[55] Tidke S.A., Devappa R., Vasist K.S., Kosturkova G.P., Gokare R.A. (2018). Soybean plants treated with vesicular arbuscular mycorrhiza fungi exhibit enhanced plant growth and nutraceutically important metabolites. J. Plant Sci..

[56] Gerdemann J.W., Nicolson T.H. (1963). Spores of mycorrhizal Endogone species extracted from soil by wet sieving and decanting. Trans. Br. Mycol. Soc..

[57] Jenkins W.R. (1964). A rapid centrifugal-flotation technique for separating nematodes from soil. Plant Dis. Rep..

[58] Schenck N., Pérez Y. (1987). Manual for the Idenfication of VA Mycorrhizal Fungi.

[59] (2018). International Culture Collection of Arbuscular and Vesicular-Arbuscular Mycorrhizal Fungi—INVAM. http://invam.wvu.edu/methods/cultures/single-species-cultures.

[60] Kraus J.E., Arduin M. (1997). Manual básico de métodos em morfologia vegetal.

[61] Johansen D.A. (1940). Plant Microtechnique.

[62] Gerlach G. (1969). Botanische Mikrotechnik.

[63] Adams R. (2007). Identification of Essential Oil Components by Gas Chromatography/Mass Spectrometry.

[64] NIST. National Institute of Standard and Technology NIST Standard Reference Database Number 69. http://webbook.nist.gov/.

[65] Mondello L. (2011). Flavors and Fragrances of Natural and Synthetic Compounds 2.

[66] Meireles E.N., Xavier L.P., Ramos A.R., Maia J.G.S., Setzer W.N., da Silva J.K.R. (2016). Phenylpropanoids produced by *Piper divaricatum*, a resistant species to infection by *Fusarium solani* f. sp. *piperis*, the pathogenic agent of fusariosis in black pepper. J. Plant Pathol. Microbiol..

[67] Axelrod B., Cheesbrough T.M., Laakso S. (1981). Lipoxygenases in soybean. Methods Enzymol..

[68] Vaganan M.M., Ravi I., Nandakumar A., Sarumathi S., Sundararaju P., Mustaffa M.M. (2014). Phenylpropanoid enzymes phenolic polymers and metabolites as chemical defenses to infection of *Pratylenchus coffeae* in roots of resistant and susceptible bananas (*Musa* spp). Indian J. Exp. Biol..

[69] Sousa C.M.M., Silva H.R., Vieira-JR G.M., Ayres M.C.C., Costa C.L.S., Araújo D.S., Cavalcante L.C.D., Barros E.D.S., Araújo P.B.M., Brandão M.S. (2007). Fenóis totais e atividade antioxidante de cinco plantas medicinais. Quím. Nova.

